# Supplementary material for: Understanding the relationships between 24-hour movement behavior, community mobility and the neighborhood built environment for healthy aging in Brazil: The EpiMove study protocol
Source: PLoS One. 2024 Dec 5;19(12):e0315021. doi: 10.1371/journal.pone.0315021 (PMC11620589; doi:10.1371/journal.pone.0315021)
Supplement: S2 Table — (DOCX) [file pone.0315021.s002.docx]

| **Table S2: Subscales and Sample Items From the Neighborhood Environment Walkability Scale** | |
| --- | --- |
| **Subscales** | **Items** |
| **Proximity to shops and commerce** | Are there places like supermarkets, convenience stores/groceries, and farmers' markets near your home? |
|  | Are there places like stores, bookstores, banks, pharmacies, beauty salons, and barbershops near your home? |
|  | Are there places like restaurants, bakeries, snack bars, and cafes near your home? |
|  | Are there places like health centers and community centers near your home? |
|  | Are there bus stops near your home? |
|  | Are there public spaces like parks, squares, walking tracks, bike lanes, and/or sports courts near your home? |
|  | Are there places like gyms and/or clubs near your home? |
|  | Are there places like churches and places of worship near your home? |
|  | Are there places like beaches and green areas near your home? |
| **Access to services** | Can you do most of your shopping at local stores? |
|  | Are there several places you can easily walk to from your home? |
|  | Is it easy to walk from your home to a bus stop (bus terminal)? |
|  | Are the streets in your neighborhood steep, making it difficult to walk on them? |
|  | Are there many hills/depressions/walls in your neighborhood limiting the number of routes for getting from one place to another? |
| **Street connectivity** | Are the distances between intersections in your neighborhood generally short (less than 100 meters)? |
|  | Are there several alternative routes you can take to get from one place to another in your neighborhood (you do not always have to take the same route)? |
| **Walking/cycling facilities** | Are there sidewalks on most streets near your home? |
|  | Are the sidewalks near your home well-maintained (paved and without holes)? |
|  | Are there bike lanes and/or trails (places for pedestrians to walk) in your neighborhood that are easily accessible? |
|  | Are the streets near your home flat (without ups and downs that make walking or cycling difficult)? |
| **Neighborhood aesthetics** | Are there green areas (such as trees or flowerbeds) along the sidewalks and streets near your home? |
|  | Do the trees provide shade on the sidewalks in your neighborhood? |
|  | Are there many interesting things to look at while walking in your neighborhood? |
|  | Are there places with garbage accumulation and/or open sewage on the streets near your home? |
| **Traffic-related safety** | Does car, bus, truck, and motorcycle traffic make it difficult to walk or use a bike near your home? |
|  | Is the traffic speed on the streets near your home generally low (30 km/h or less)? |
|  | Do most drivers exceed the speed limit while driving in your neighborhood? |
|  | Are there crosswalks, signals, or overpasses that help pedestrians cross the streets near your home? |
|  | Is there a lot of noise/pollution near your home? |
| **Crime-related safety** | Are the streets near your home well-lit at night? |
|  | When walking in your neighborhood, do you talk to other people? |
|  | During the day, do you feel safe walking, cycling, or exercising near your home? |
|  | At night, do you feel safe walking, cycling, or exercising near your home? |
|  | Is there a high level of crime in your neighborhood, such as vandalism of public and private places, thefts, assaults, break-ins, assaults, etc.? |
